# How to feed small for gestational age newborns

**DOI:** 10.1186/1824-7288-39-28

**Published:** 2013-05-10

**Authors:** Giovanni Barone, Luca Maggio, Annalisa Saracino, Alessandro Perri, Costantino Romagnoli, Enrico Zecca

**Affiliations:** 1 Division of Neonatology, Department of Pediatrics, Catholic University Sacred Heart, Largo A. Gemelli 8, Rome 00168, Italy

**Keywords:** Newborn, Small for gestational age, Feeding, Nutrition

## Abstract

Feeding small for gestational age (SGA) newborns is extremely challenging and the neonatologist should be brave and cautious at the same time. Although these babies have a high risk of milk intolerance and necrotising enterocolitis, enteral feeding guidelines are not well established and practice varies widely among different neonatal units. Currently available studies on this topic include extremely and very low birth weight neonates, but are not focused specifically on small for gestational age infants. This review analyzes papers focused on feeding interventions in order to provide the best available evidences about the optimum timing for introduction of enteral feeding, how fast feed volume can be advanced, which milk and which feeding method is more appropriate in SGA infants.

## Background

The term “small for gestational age” (SGA) is used to describe newborns whose birth weight and/or crown-heel length is less than expected for their gestational age and sex. Traditionally, the term SGA has been used to describe a neonate whose weight and/or length at birth is at least 2 SD below the mean for the infant’s gestational age, equivalent to the 2.3 percentile, based on the data derived from an appropriate reference population [1]. Some publications define SGA newborns as those with birth weight or length below the 3rd, 5th, or 10th percentiles for gestational age [2]. The first definition was chosen by the international SGA advisory panel because it likely includes the majority of patients with impaired fetal growth.

However, this definition of SGA is inaccurate because it is not able to exclude the constitutional smallness, which is not pathological [3]. The term intrauterine growth retardation (IUGR) suggests diminished growth velocity in the fetus as documented by at least 2 intrauterine growth assessment. Therefore SGA and IUGR are not synonymous. IUGR indicates the presence of a pathological process occurring in utero that inhibits fetal growth. Being born SGA does not necessarily mean that an intrauterine growth retardation has occurred and infants who are IUGR are not inevitably SGA at birth.

There are several causes for being SGA. Exposure of the fetus to toxins (smoking, alcohol, drug abuse), chromosomal anomalies (trisomy 13, Edward Syndrome, Turner Sydrome, Prader-Willy Syndrome etc.), congenital infections (toxoplasmosis, rubella, cytomegalovirus), metabolic disorders, maternal factors (both young and advanced age, maternal hypertension, placental and uterine abnormalities etc.). However the most common etiology of being born SGA is placental insufficiency that impairs growth particularly during the last trimester of pregnancy leading to IUGR [4-6]. Below we discuss the most severe and important complication for these neonates, with pathophysiological considerations.

### NEC

Necrotizing enterocolitis (NEC) is a severe inflammatory disorders in which prematurity and enteral feeding seems the major predisposing factors. It occurs in up to 7% of very low birth weight infants, with a mortality rate of 15 - 30%, inversely related to birth weight and gestational age [7]. Garite et al. in a retrospective study including 29.916 premature newborns found that both SGA and IUGR were independently associated with an increased risk of NEC [8] By the physiological point of view growth restriction modifies the developmental pattern of intestinal structure. The intestine of SGA neonates has reduced weight, length, wall thickness, villous weight, and crypt depth [9,10]. Furthermore these infants have intestinal dysbiosis and an alteration of the proliferation-apoptosis homeostasis which leads to a reduced surface of intestinal exchange [11]. These alterations could be responsible for the higher gastrointestinal morbidity, feeding intolerance and impaired nutrient absorption.

However recently much attention was focused on those infants born prematurely with IUGR and abnormal blood flow on antenatal Doppler studies [12]. Increased placental resistance in the presence of placental failure leads to a reduction in end diastolic blood flow through the umbilical arteries, progressing to absent (AEDF) or reversed flow (AREDF) [13]. Pathophysiology of fetal adaptation to chronic hypoxia involves preferential shunting of blood to the brain at the expense of the splanchnic circulation. It was shown that severe prenatal Doppler abnormalities are associated with poor fetal outcome [14,15], but it is still debated if they increased the risk of neonatal NEC.

Some studies have demonstrated a close association between AEDF or AREDF and NEC, which appears to be independent of other factors such as degree of growth retardation, prematurity and perinatal asphyxia [16,17], while others have not confirmed these findings [18,19]. A meta-analysis of 14 observational studies demonstrated an increased incidence of NEC in preterm infants who had suffered fetal AREDF compared with controls, with an odds ratio of 2.13 (95% CI 1.49 to 3.03) [20]. Nine of the included studies showed an excess of NEC in the AREDF infants; eight studies classified NEC using the stricter definition of radiological or surgical confirmation, of which six showed an excess of confirmed NEC in the AREDF group. Overall, confirmed NEC was not significantly increased in these studies (OR 1.6, 95% CI 0.9 to 2.8), but the six studies examining confirmed NEC in preterm infants with IUGR showed greatly increased odds of confirmed NEC in infants with fetal AREDF (OR 6.9, 95% CI 2.3 to 20). In many of the studies, fetuses with AREDF required earlier delivery than controls so it could be argued that the higher risk of NEC in these studies was primarily related to the lower gestational age and birth weight; nevertheless, the excess of confirmed NEC was also found in the two series that matched controls for gestation and weight (OR 5.5, 95% CI 1.1 to 28) [16,21]. A more recent study confirmed the results of this meta-analysis demonstrating a strong relation between AREDF and subsequent development of NEC (OR: 5.88, 95% CI: 2.41 to 14.34) also after adjustment for gestational age at birth (OR: 7.64, 95% CI: 2.96 to 19.70,) and after adjustment for birth weight for gestational age z-score (OR: 6.72, 95% CI: 2.23 to 20.25) [22].

All the previous studies examined only the role of umbilical arteries Doppler flows. When Manogura et al. [23] investigated a more comprehensive fetal Doppler assessment that provided greater circulatory details (umbilical artery, middle cerebral artery, ductus venosus, and umbilical vein) the association between NEC and AREDF was lost. In this study, a multinomial logistic regression with NEC as dependent variable failed to demonstrate a relationship between placental resistance and the risk of NEC, and found that birth weight and base deficit at birth were the independent risk factors for NEC. These results have raised some doubts on the reliability of all the evidences suggesting a causal relationship between NEC and abnormal placental resistance. Moreover, many studies were underpowered given the overall low incidence of NEC, and the metabolic status at birth was not taken into consideration by any of these studies. If it is plausible that placental insufficiency predisposes to, but does not initiate, the cascade of events that lead to NEC, it is more likely that the limitations of prematurity define the origins of this disease.

### The questions about feeding SGA infant

Enteral feeding guidelines are not well established in preterm SGA neonates, and there is a lack of published information about best feeding regimen. Practice varies widely among different neonatal units as shown by a survey carried out in two different English Health Regions, but a policy of delayed and careful introduction of enteral feeding is often chosen in order to prevent NEC [20]. We now analyze the best current evidences on feeding SGA infants.

### What milk

Human breast milk would be expected to protect against NEC for its antimicrobial and anti-inflammatory characteristics. However, proving efficacy in randomized clinical trial has been challenging because of 2 main reasons. First of all, the difficulty of recruiting infants to a randomized trial about human milk when mothers have strong preferences, secondly the lack of standardized definitions of what human milk comprises (maternal or donor, fortified or unfortified, human milk alone or human milk plus formula).

In 1990 Lucas and Cole demonstrated a reduction in the incidence of NEC among preterm infants who received only human milk when compared with infants who received bovine milk–based formula [24]. Two meta-analysis of several small randomized controlled trials reported a lower incidence and severity of NEC in infants fed with an exclusively human milk diet [25,26]. A recent trial randomized 207 premature infants with a birth weight between 500 and 1250 grams to receive fortified human milk or bovine-milk based products and confirmed earlier data finding that the rates of NEC and NEC requiring surgery were markedly lower in the first group. The number of infants needed to treat (NNT) with an exclusively human milk diet to prevent 1 case of NEC was 10 and NNT to prevent 1 case of surgical NEC or death is 8 [27].

### Early vs delayed

Early enteral feeding is advantageous because it improves the functional adaptation of the gastrointestinal tract by stimulating hormone secretion and gastrointestinal motility [28]. It also decreases the need of total parenteral nutrition and its associated complications, such as catheter related sepsis, cholestasis, cardiac tamponade, osteopenia of prematurity and other metabolic disturbances [29,30]. Despite this, early enteral feeding is often delayed in high risk infants because it has been thought to be associated with an increased risk of NEC. A meta-analysis of five RCTs conducted on preterm infants did not detect a significantly different risk of NEC between infants randomized to delayed feeding (defined as introduction of enteral feeds as later than day 5–7 after birth) and infants randomized to early feeding (defined as less than 4 day after birth); RR 0.89 (95% CI 0.58 to 1.37) [31]. The two largest trials in that meta-analysis [32,33] recruited only SGA infants with abnormal fetal circulatory distribution or flow. For these reasons, data from these trials do not provide sufficient evidence that delayed introduction of enteral feeding in SGA neonates reduces the risk of NEC, even if 95% CI for the pooled estimates of effect is wide and consistent with more than 40% reduction in the risk of NEC and death in newborns who have delayed introduction. Given this level of uncertainty this findings should be applied cautiously.

### Minimal enteral feeding

An alternative approach to delaying feeding is the minimal enteral feeding (MEF). MEF (also known as “trophic feeding”, “gut-priming”, “non nutritive feeding” and “hypocaloric feeding”) is conventionally defined as giving small volumes of milk (typically 12 to 24 ml/kg/day every 1–3 hours) starting within the first few days after birth without advancing the feed volumes during the first week of life [34]. Enteral fasting during the early neonatal period has potential disadvantages for premature infants, because gastrointestinal hormone and motility are improved by enteral milk. Delayed enteral feeding could impair the functional adaptation of the gastrointestinal tract leading to intestinal dismotility and consequent feeding intolerance [35,36]. A systematic review published in the Cochrane Library [37] did not detect a statistically significant effect on the incidence of NEC between very low birth weight newborns randomized to MEF and to no enteral feeding (RR 1.07 95% CI 0.67 to 1.70). Substantial clinical uncertainty remains about the effect of MEF on SGA infants because most of the trials on this topic specifically exclude infants who were SGA at birth. The only one including 56 babies with birth weight below 2000 grams and below 10th percentile for gestational age failed to demonstrate significant differences between newborns fed with trophic feeding or no feeds for the first five days of life in the primary outcome of intestinal permeability measured by the sugar absorption test [38]. There were also no differences in feeding tolerance, growth and incidence of NEC between the two groups [38].

### How to advance feed volume

The rate of advancement of enteral feeding is another area of uncertainty. Retrospective studies have found that those neonatal centers where enteral feeding is introduced earlier and feeding volume advanced faster have higher incidences of NEC [39]. On the other hand, slow advancement of enteral feeding delays the establishment of full enteral nutrition and extends the duration of total parenteral nutrition with its associated risks [40], that may have adverse consequences for survival, growth and development [41]. A meta-analysis of four trials (496 very low birth weight infants) showed no differences in NEC rates comparing rapid (as 30 to 35 ml/kg/day) versus slow (as 15 to 20 ml/kg/day) advancement feeding strategies (RR 0.91 95% CI 0.47 to 1.75) [42]. Infants fed at faster rate reached the full enteral feeding about two to five days earlier than infants fed slowly, but they did not have a higher risk of NEC. However, these findings should be applied with caution to SGA newborns because the vast majority of the studied infants were appropriate for gestational age. Only in the trial performed by Salhotra et al. [43] more than 95% of the 53 participants were SGA. In this trial, the fast enteral feeding group reached the full enteral feed significantly earlier (mean 10 days) than the slow advancement group (mean 14.8 days), and there were two cases of NEC in the fast advancement group. To date there are no trial that compare slow versus fast feeding regimen in a selected population of SGA newborns.

### Mode of feeding

To date there are no studies focused on SGA newborns and the best mode of feeding. The few available data concern premature infants born < 1500 grams that are not able to coordinate sucking, swallowing, and breathing. A systematic review of seven trials published in the Cochrane Library [44] did not detect a statistically significant effect between continuous versus intermittent milk feeding methods in time to achieve full enteral feeding, in feeding intolerance, in somatic growth and in incidence of NEC. At the present time practice appears to be based more on individual assessment rather than on scientific evidence. Continuous feeding may reduce energy expenditure [45] and improve feeding tolerance, nutrient adsorption and growth [46]; on the other hand, intermittent bolus method may be more physiologic, promoting the cyclical pattern of release of gastrointestinal hormones, which are important for gut development [47].

### Feeding intolerance

Feeding intolerance is usually characterized by gastric residuals before feeding, emesis and abdominal distention. The gastric residual volume (GRV) is the element of feeding that can be measured and compared most easily. Several authors suggested to use GRV as a marker of feeding intolerance, in order to make early detection of NEC [48-50]. Qualitative and quantitative evaluation of gastric residuals can be performed. To date is difficult to assess a tolerance threshold of GRV beyond which enteral feeding should be withdrawn. Mihatsch et al. [48] tolerated GRV up to 2 mL in newborns ≤ 750 grams and up to 3 mL in newborns from 750 to 1000 grams in their protocol, but concluded that additional research is required to evaluate if GRV threshold could be increased up to 5 ml/kg body weight. Cobb et al. [49] found that GRV > 3.5 mL or 33% of a single meal may be associated with a higher risk for NEC while a GRV <1.5 mL or 25% of a meal is probably normal. Finally the available data on qualitative evaluation of gastric residuals suggest that infants with blood stained or hemorrhagic residuals were at higher risk of NEC, whereas bile stained residuals are not a risk factor by themselves [50].

### Quality of the evidence

Being born SGA does not necessarily mean that IUGR has occurred, and infants who are IUGR are not inevitably SGA at birth. Unfortunately, the terms IUGR and SGA have been used interchangeably, creating confusion on the topic. In the absence of congenital malformations or chromosomal abnormalities, small fetal size could be the consequence of two distinct processes: constitutional smallness or pathological growth restriction. Distinguishing one process from the other is challenging, but such distinctions have profound implications toward understanding quality and robustness of evidence provided by available trials. Patients enrolled in the studies are usually selected according to their birth weight (below the 10th or the 3rd percentile) without checking if a growth restriction really occurred. So SGA is a term that is often used as a proxy for restricted growth, thereby combining both constitutionally small and pathologically growth restricted fetuses. It is known that growth restricted fetuses are small because of some underlying pathological conditions (smoking during pregnancy, uteroplacental dysfunction, hypertensive disorders, etc.), and they are therefore at increased risk for neonatal morbidity and mortality. On the other hand, constitutionally small infants can easily have morbidity and mortality very similar to appropriate for gestational age, and considerably lower than pathologically growth restricted ones [51].

## Conclusions

There is limited evidence on which to base feeding policy in SGA newborns. Currently available studies on this topic include extremely and very low birth weight neonates, but are not focused specifically on SGA infants. Furthermore there are not RCTs that make a clear distinction between SGA and growth restricted neonates. Future randomized trials on feeding intervention should be targeted on IUGR infants, excluding constitutionally small newborns, in order to provide robust evidence concerning the optimum timing for introduction of enteral feeding, how fast feed volume can be advanced and which feeding method is more appropriate. To date, however, no trials showed any benefits of delayed enteral feeding or slow advancement of enteral feed volumes. Growth restricted newborns are a nutritional emergency that will result in serious short and long term detrimental effects, when left untreated.

## Abbreviations

SGA: Small for gestational age; IUGR: Intrauterine growth restriction; NEC: Necrotizing enterocolites; AEDF: Absent end diastolic flow; AREDF: Absent and reverse end diastolic flow; SMA: Superior mesenteric artery; MEF: Minimal enterale feeding; GRV: Gastric residual volume.

## Competing interests

The authors declare that they have no competing interests.

## Authors’ contributions

GB and EZ conceived the study and has made substantial contributions in drafting manuscript. LM and AS performed literature review and made contributions in drafting manuscript. CR revised the manuscript critically. AP gave a valuable contribution to the revision of the manuscript according to reviewer’s comments. All authors read and approved the final manuscript.
